# MALDI-TOF mass spectrometry and high-resolution melting PCR for the identification of *Mycoplasma bovis* isolates

**DOI:** 10.1186/s12917-021-02870-5

**Published:** 2021-04-17

**Authors:** Aric J. McDaniel, Rachel J. Derscheid

**Affiliations:** Departments of Veterinary Microbiology and Preventative Medicine (McDaniel) and Veterinary Diagnostic and Production Animal Medicine (Derscheid), College of Veterinary Medicine, Iowa State University, Ames, IA 50011 USA

## Abstract

**Background:**

*Mycoplasma bovis* is an important pathogen of cattle worldwide. Many different clinical manifestations of infection can occur, including respiratory disease, arthritis, and mastitis, causing heavy losses to beef and dairy industries. Because *Mycoplasma* species are slow-growing and fastidious, traditional identification methods are not cost- or time-effective, and improved methods are sought to streamline laboratory processes. High-resolution melting PCR (HRM-PCR) and matrix-assisted laser desorption/ionization time-of-flight mass spectrometry (MALDI-TOF MS) are 2 relatively recent tools that are rapid and inexpensive to use; we tested 9 isolates of *M. bovis* using both assays. The HRM-PCR assay used universal mycoplasma primers for the 16S–23S intergenic spacer region (IGSR).

**Results:**

The resulting melting profiles of the field isolates were indistinguishable from the reference strain, indicating accurate identification. For the MALDI-TOF MS, each *M. bovis* isolate was accurately identified. *Mycoplasma arginini* and *Mycoplasma alkalescens* isolates did not identify as *M. bovis* when tested by either assay.

**Conclusions:**

Our work shows that either assay could be used to identify unknown *M. bovis* isolates. For future work, the MALDI-TOF MS library should be expanded to include more mycoplasmas, and the HRM-PCR assay should be tested on additional mycoplasmas to ensure that the melting profiles are sufficiently distinctive.

## Background

*Mycoplasma bovis* is an important pathogen to the beef and dairy industries, causing respiratory disease, arthritis, mastitis, and other infections as well [1]. Few studies of its economic impact have been conducted, but it is estimated that in the United States up to $32 million per year is lost by the beef industry as a result of reduced weight gain, and up to $108 million per year is lost by the dairy industry because of mastitis [1]. Identification of *M. bovis* and other *Mycoplasma* species in general can be arduous. Although biochemical tests are the gold standard for conventional bacteria, these methods take weeks to detect the fastidious and slow-growing mycoplasmas*.* Classically, serological tests have been used to identify mycoplasma isolates [2], however these assays require many different reagents and can be cumbersome to perform. The advent of PCR has been critical to increase the detection of *Mycoplasma* species, with the benefit of being able to perform PCR on field samples without prior culture. Drawbacks to PCR include the questionable clinical relevance of high Cts, the potential for missing some strains depending on the assay [3], and the inability to identify co-infections or infections with other *Mycoplasmas.* To quickly diagnose cattle to prevent further spread of the infection and facilitate selection of appropriate treatment, faster methods of identification are needed.

Matrix-assisted laser desorption/ionization-time of flight mass spectrometry (MALDI-TOF MS) is a tool that can be used to analyze the protein composition of bacteria [4]. In the last 15 years, this technology has been used to identify bacteria based on their protein profile [5]. With regards to *Mycoplasma* species, MALDI-TOF MS has been used to identify clinically relevant human isolates [6, 7] as well as isolates from a variety of animal species, including *M. bovis* from cattle [6, 8–11].

Another laboratory tool is high-resolution melting PCR (HRM-PCR). This technology can be used for a variety of purposes, such as genotyping and strain differentiation of bacteria, [12, 13] and identification of viruses and fungi [14, 15]. One benefit of HRM-PCR over traditional real-time PCR for pathogen identification is that one set of primers can be used to distinguish between different species or different strains of the same species, increasing efficiency. Depending on the target for the assay, different species or strains will exhibit a unique melting profile. Much of the HRM-PCR work done with *Mycoplasma* spp. has been with poultry isolates to distinguish between vaccine strains and field strains of the same species [16–18]. Experiments with general strain differentiation have been performed as well [19–21] in addition to one study using HRM to detect mutations conferring antibiotic resistance in *Mycoplasma hyopneumoniae* [22]. Little HRM-PCR has been done for identification of *Mycoplasma* spp., although the efficacy of such an assay using universal primers for canine, avian, and ruminant mycoplasmas has been explored [23].

We compared the accuracy of HRM-PCR and MALDI-TOF MS in the identification of 8 *M. bovis* strains isolated at the Iowa State University Veterinary Diagnostic Laboratory and identified by species-specific PCR [24] along with a reference strain. Our research assesses two emerging diagnostic tools to further improve the diagnostic process for identification of *Mycoplamsa bovis* isolates.

## Results

**Fig. 1:**
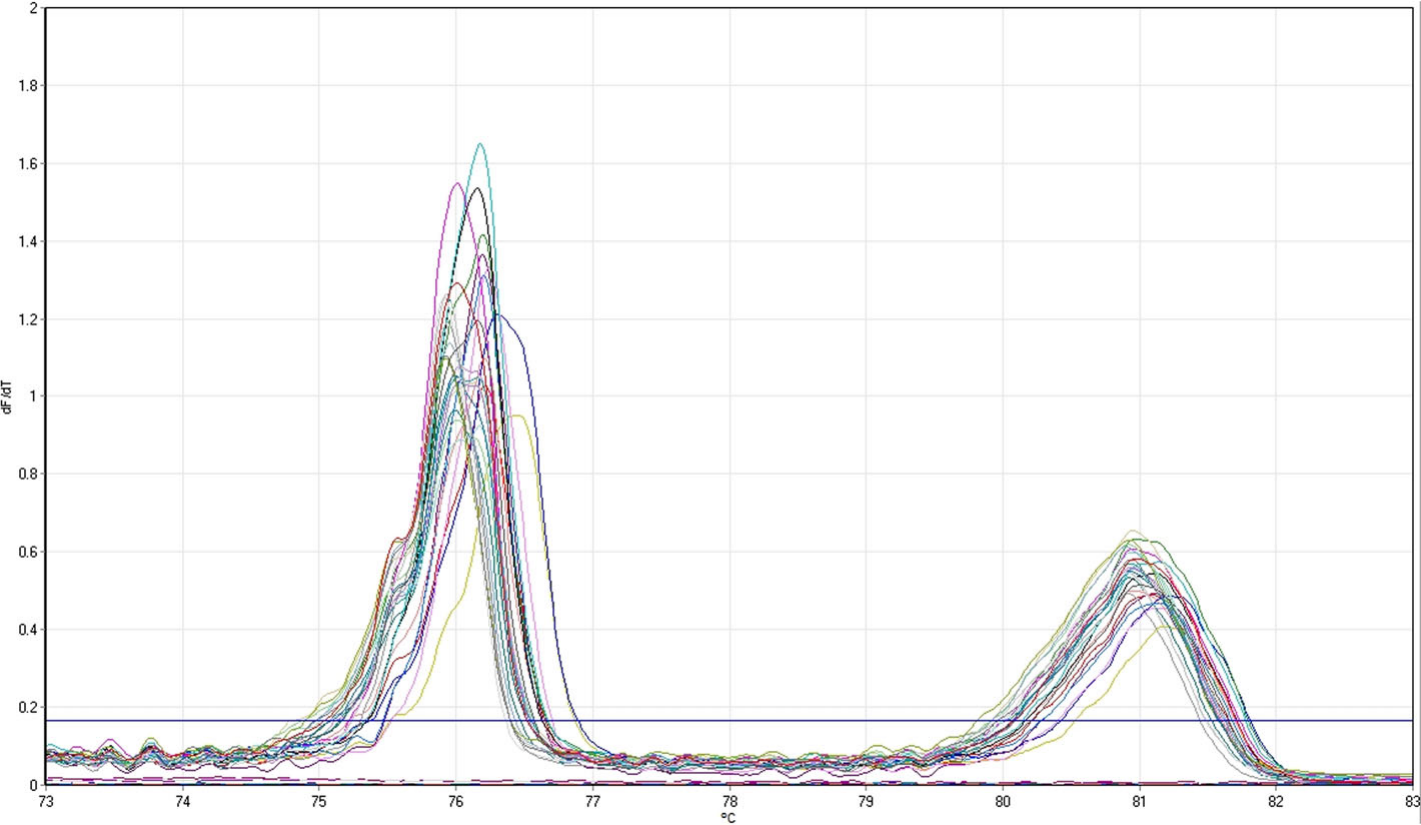
Identification of *Mycoplasma bovis* isolates by high-resolution melting PCR. HRM-PCR was done in triplicate for each isolate. The PCR threshold was determined automatically by the Qiagen Rotor-Gene Q Series Software

The 8 *M. bovis* isolates and the *M. bovis* reference strain exhibited 2 melt peaks: the first at 76.25 ± 0.005 °C and the second at 80.94 ± 0.008 °C. Additionally, the height of the second peak was one-third the height of the first peak (Fig. 1). No significant difference between the melting temperatures was present when comparing the field strains to the reference strain for either the first peak or the second peak (Table 1).

**Table 1:** Mean melt peak temperatures^a^ for *Mycoplasma bovis* isolates

Isolate	Mean temperature (°C)	
	Peak 1	Peak 2
B1	76.255 ± 0.001	80.941 ± 0.001
B2	76.259 ± 0.005	80.950 ± 0.008
B3	76.257 ± 0.004	80.930 ± 0.005
B4	76.251 ± 0.003	80.940 ± 0
B5	76.256 ± 0.002	80.938 ± 0.002
B6	76.253 ± 0.002	80.945 ± 0.003
B7	76.251 ± 0.001	80.933 ± 0.001
B8	76.245 ± 0.001	80.937 ± 0.002
B9	76.249 ± 0.001	80.937 ± 0.001
*M. alkalescens*	76.083 ± 0.010	82.858 ± 0.004
*M. arginini*	76.082 ± 0.017	82.712 ± 0.013

^a^Data represents the mean of the 3 replicates ± SE

**Fig. 2:**
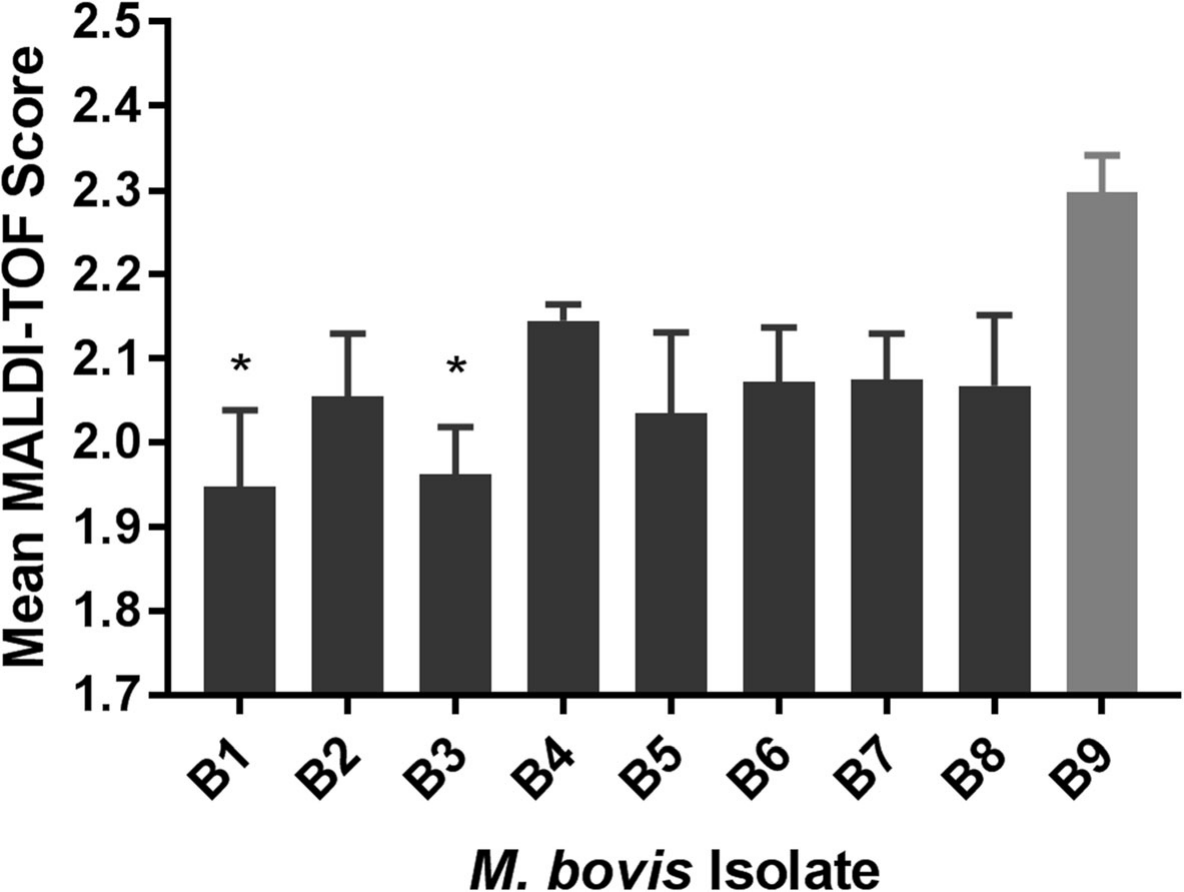
Mean MALDI-TOF MS score of the four spots for each *Mycoplasma bovis* isolate. The Dunnett test was performed comparing the field isolates to B9 (gray), the reference strain. An asterisk indicates *p* ≤ 0.05

None of the additional bovine mycoplasmas exhibited a second peak of ~ 80.94 °C and were not identified as *M. bovis*. Every *M. bovis* isolate was correctly identified as *M. bovis* by the MALDI-TOF MS software, although the top scores ranged from 1.71 to 2.39. The reference strain (ATCC25025, B9) had the highest score, but it was only significantly higher than 2 of the 8 field strains (Fig. 2) The other two species both had a result of no organism identification possible, indicating that there was no match in the library to the spectra that were generated.

## Discussion

In the HRM-PCR assay, 2 melting peaks were present for each *M. bovis* isolate, at temperatures consistent with previous findings for this assay [23]. A possible explanation for the 2 peaks is that the *M. bovis* genome contains 2 copies of the rRNA genes, and if the sequences vary sufficiently, they could have resulted in different melting temperatures (Tm) [25]. When comparing the DNA sequences of the 2 16S–23S IGSR and the segments of the 16S and 23S rRNA that would be amplified by this assay, the sequences are almost identical identical in *M. bovis* strain PG45. The non-IGSR parts of the amplicons are exactly the same, and only 2 deletions and 1 single nucleotide polymorphism are present in the second copy compared to the first copy. This would likely not be enough to change the Tm by almost 5 °C, and even if it would, the brightness of the 2 peaks would be approximately equal given that the 2 16S–23S IGSR sequences are both 311 bp in length [25]. An alternative explanation for the presence of 2 peaks is that the sequences have 2 regions with significantly different GC content, resulting in a partial melt followed by a complete melt at a higher temperature. This second explanation seems to be more likely, as the second, higher Tm (80.94 °C) of each isolate was less fluorescent, indicating that less dye was released and so less DNA remained to melt.

The MALDI-TOF MS scores for the reference strain of *M. bovis* tended to be higher than at least some of the field strains. This was expected, given that spectra for that strain are present in the MALDI-TOF MS library that we used. Although the MALDI-TOF MS software identified each *M. bovis* isolate correctly, spectra from only 10 *Mycoplasma* spp. other than *M. bovis* were present. None of these were from closely related species such as *M. agalactiae*, *M. californicum*, or *M. fermentans* [26, 27]. It is worth noting, however, that none of the field isolates were misidentified as one of the other mycoplasmas present in the library, nor were the other mycoplasmas misidentified as *M. bovis*. Many of the MALDI scores were also between 1.7 and 2.0, indicating that the top scoring organism may not be acceptable for species-level identification, though other investigators have determined that scores > 1.7 may be sufficient for certain *Mycoplasma* spp. including *M. bovis* [6].

HRM-PCR and MALDI-TOF MS are testing different aspects of the genetic code, and depending on the circumstance, one test may be preferred. MALDI-TOF MS creates spectra from the proteins of the cultures whereas HRM-PCR generates a melt curve profile from the DNA. MALDI-TOF MS would find no difference between 2 isolates with varying silent mutations, because these do not affect the encoded amino acids, and for identification purposes, this would be preferred. As new, variant isolates of a species are discovered, additional spectra can be added to the database to improve its identification capabilities. HRM-PCR relies on only one isolate per species as a control, whereas MALDI-TOF MS needs spectra from multiple isolates of a species in order to get a well-rounded library of spectra of a given species. If the primer target sequence varies too much with a given species however, HRM-PCR will not be able to identify that organism, therefore the PCR primers must be extremely well-validated.

In the future, other mycoplasmas should be explored for the HRM-PCR assay to confirm that between *Mycoplasma* species the melt curve profile generated is unique, but within species it is the same, i.e., determine the specificity and sensitivity, respectively. Additionally, the potential use of HRM-PCR directly of tissue samples should be investigated, as this would drastically decrease the time and cost required for identification. For MALDI-TOF MS, the addition of spectra of species more closely related to *M. bovis* may be beneficial; however, additional reference strains are vital for more consistent and accurate identification. Focus should be given also to the isolates identified in our study as *M. bovis* but with scores between 1.7 and 2.0, because these are more likely to improve the library for this organism as they have been shown to be less similar to the isolates currently in the library.

## Conclusion

The 2 methods that were explored both resulted in accurate identification 100% of the time for the *M. bovis* isolates utilized in our study. The HRM-PCR assay found no difference between the melting peaks of the field isolates and the reference strain, and all replicates of the *M. bovis* isolates in MALDI-TOF MS resulted in scores of ≥1.7. Both assays can be completed in less than 2 h and, if using validated, universal primers for the HRM-PCR, both assays could be used for many different species, making them overall much less expensive than a separate assay for each individual organism.

## Methods

Eight isolates of *Mycoplasma bovis* and one reference strain (Table 2) were grown for 3–5 days in PPLO broth with horse serum (University of California-Davis, Davis, CA) and supplemented with 5 μg/mL cefoperazone inhibitor (MilliporeSigma, Burlington, MA) for the HRM-PCR. They were then subcultured onto PPLO agar with horse serum (University of California-Davis) and grown for 4 days for use in MALDI-TOF MS. All isolates were grown at 37 °C with 7.5% CO_2_. In order to evaluate the specificity of the assays, *M. arginini* and *M. alkalescens*, which are frequently isolated from cattle [28] and were identified by sequencing of the 16S rRNA gene, were grown as stated above and tested with both assays.

**Table 2:** *Mycoplasma* isolates and their origin

Isolate	Animal host	Site of isolation	Year isolated	State of origin
1	Cattle	Joint fluid	2013	Iowa
2	Cattle	Ear swab	2013	Missouri
3	Cattle	Lung	2014	Iowa
4	Cattle	Bulk tank milk	2014	Iowa
5	Cattle	Lung	2014	Kentucky
6	Cattle	Joint fluid	2014	Iowa
7	Cattle	Eye swab	2015	Iowa
8	Bison	Lung	2016	Iowa
9 (ATCC 25025)	Cattle	Milk	1966	Connecticut
*M. alkalescens*	Cattle	Joint	2019	Iowa
*M. arginini*	Cattle	Eye swab	2018	Iowa

All 8 strains were isolated from ruminants from different locations in the United States

Genomic DNA was extracted from 200 μL of liquid culture by boiling for 10 min. The cells were then pelleted by centrifugation in a tabletop centrifuge at 15,871×g for 3 min. In order to mimic the high-throughput environment of a diagnostic laboratory, the amount of DNA in each extract was not standardized. Universal primers targeting the 16S–23S intergenic spacer region (IGSR) were used for this assay as previously described: F 5′-ACACCATGGGAGYTGGTAAT-3′ and R 5′-CTCCWTCGACTTYCAGACCCAAGGCAT-3′ [23]. The Qiagen HRM PCR Master Mix (Qiagen, Venlo, Netherlands) was used according to manufacturer’s recommendations with 2.5 μL of DNA extract per well. Sterile water was used in place of the extract for the negative amplification control, and sterile medium was extracted for the negative extraction control. The HRM-PCR was performed in triplicate (RotorGene Q; Qiagen) using the cycling conditions described previously [23].

After growing for 4 days on solid medium, the 8 isolates and the *M. bovis* reference strain were run in MALDI-TOF MS (MALDI BioTyper 3.1; Bruker Daltonics, Billerica, MA) using the reference database MBT 6903 MSP Library (829023). The library includes spectra for the following *Mycoplasma* species (number of isolates)*: M. alkalescens* (2)*, M. arginini* (2)*, M. bovis* (2)*, M. gallinaceum* (2)*, M. gallisepticum* (2)*, M. hominis* (2)*, M. ovipneumoniae* (2)*, M. pullorum* (2)*, M. canis* (1)*, M. hyorhinis* (1)*,* and *M. salivarium* (1). Two additional spectra for *M. bovis* were added by the ISUVDL, one of which was the reference strain (ATCC 25025). As the colonies were small, multiple colonies of each isolate were directly spotted from the agar plates 4 times onto a single steel target using a sterile loop. α-Cyano-4-hydroxycinnamic acid (HCCA) was applied as matrix and the isolates were then run in the instrument according to the manufacturer’s recommendations (Bruker Daltonics).The result is expressed as a logarithmic score of 0 to 3 with a score of ≥2.0 considered acceptable for identification at the species level. This score is based on the matches and intensity of the peaks detected (MALDI BioTyper 3.1 User Manual. 2012. Bruker Daltonics).

Using the whole genome sequence of *Mycoplasma bovis* PG45 (ATCC 25523) available in GenBank under accession CP002188, the sequences of the 2 copies of the 16S–23S intergenic spacer regions 16S rRNA, and 23S rRNA that would be amplified by the HRM-PCR assay were analyzed and compared. These two sequences were aligned using MEGA-X to observe differences in the sequences. The Dunnett test for multiple comparisons was used to compare the melting temperatures of the field isolates to those of the reference strain for the HRM-PCR assay. MALDI-TOF MS data was analyzed using a one-way ANOVA with the Tukey multiple comparison test.

## Data Availability

The 16S–23S intergenic spacer region genome sequences are available in GenBank under accession CP002188. Any datasets used or analyzed that are not clearly included in the text are available from the corresponding author upon request.

## Abbreviations

HRM-PCR: High resolution melting curve polymerase chain reaction
MALDI-TOF MS: Matrix-assisted laser desorption/ionization time-of-flight mass spectrometry
IGSR: Intergenic spacer region
